# Change in habitual intakes of flavonoid-rich foods and mortality in US males and females

**DOI:** 10.1186/s12916-023-02873-z

**Published:** 2023-05-12

**Authors:** Nicola P. Bondonno, Yan Lydia Liu, Yan Zheng, Kerry Ivey, Walter C. Willett, Meir J. Stampfer, Eric B. Rimm, Aedín Cassidy

**Affiliations:** 1 Institute for Global Food Security, Queen’s University Belfast, Belfast, Northern Ireland; 2 Nutrition and Health Innovation Research Institute, School of Medical and Health Sciences, Edith Cowan University, Perth, Australia; 3 Danish Cancer Society Research Centre (DCRC), Copenhagen, Denmark; 4 Department Nutrition, Harvard T.H. Chan School of Public Health, Boston, MA USA; 5 State Key Laboratory of Genetic Engineering, School of Life Sciences and Human Phenome Institute, Fudan University, Shanghai, China; 6 Channing Division of Network Medicine, Brigham and Women’s Hospital and Harvard Medical School, Boston, MA USA; 7 Department of Epidemiology, Harvard T.H. Chan School of Public Health, Boston, MA USA

**Keywords:** Flavonoids, All-cause mortality, Flavodiet score

## Abstract

**Background:**

Higher baseline intakes of flavonoid-rich foods and beverages are associated with a lower risk of chronic disease and mortality in observational studies. However, associations between changes in intakes and mortality remain unclear. We aimed to evaluate associations between 8-year changes in intakes of (1) individual flavonoid-rich foods and (2) a composite measure (termed the ‘flavodiet’) of foods and beverages that are known to be main contributors to flavonoid intake and subsequent total and cause-specific mortality.

**Methods:**

We evaluated associations between 8-year changes in intakes of (1) individual flavonoid-rich foods and (2) a novel ‘flavodiet’ score and total and cause-specific mortality. We included 55,786 females from the Nurses’ Health Study (NHS) and 29,800 males from the Health Professionals Follow-up Study (HPFS), without chronic disease at baseline in our analyses. Using multivariable-adjusted Cox proportional hazard models, we examined associations of 8-year changes in intakes of (1) flavonoid-rich foods and (2) the flavodiet score with subsequent 2-year lagged 6-year risk of mortality adjusting for baseline intakes. Data were pooled using fixed-effects meta-analyses.

**Results:**

We documented 15,293 deaths in the NHS and 8988 deaths in HPFS between 1986 and 2018. For blueberries, red wine and peppers, a 5%, 4% and 9% lower risk of mortality, respectively, was seen for each 3.5 servings/week increase in intakes while for tea, a 3% lower risk was seen for each 7 servings/week increase [Pooled HR (95% CI) for blueberries; 0.95 (0.91, 0.99); red wine: 0.96 (0.93, 0.99); peppers: 0.91 (0.88, 0.95); and tea: 0.97 (0.95, 0.98)]. Conversely, a 3.5 servings/week increase in intakes of onions and grapefruit plus grapefruit juice was associated with a 5% and 6% higher risk of total mortality, respectively. An increase of 3 servings per day in the flavodiet score was associated with an 8% lower risk of total mortality [Pooled HR: 0.92 (0.89, 0.96)], and a 13% lower risk of neurological mortality [Pooled HR: 0.87 (0.79, 0.97)], after multivariable adjustments.

**Conclusions:**

Encouraging an increased intake of specific flavonoid-rich foods and beverages, namely tea, blueberries, red wine, and peppers, even in middle age, may lower early mortality risk.

**Supplementary Information:**

The online version contains supplementary material available at 10.1186/s12916-023-02873-z.

## Background

Findings from prospective cohort studies, supported by recent randomised controlled trials, provide growing evidence that higher intakes of plant-based foods and beverages, rich in bioactive compounds such as flavonoids, may offer distinct benefits in relation to health and the prevention of premature death [1–4]. This is attributed to the role of flavonoids in protecting against many chronic diseases, as demonstrated by converging evidence from in vitro, clinical and epidemiological studies [5, 6]. Flavonoids are the most abundant and most researched class of polyphenols, a major group of phytochemicals which demonstrate important biological effects [7]. Although nearly all plant-based foods contain flavonoids, some contain uniquely high concentrations, namely tea, apples, berries, citrus fruits, dark chocolate and red wine.

Many studies on habitual food intake and health are derived from a single, baseline dietary intake measure; repeated measures of intake are preferable as benefits are likely cumulative for most chronic disease and more up-to-date consumption level data captures within-person variability over the adult lifetime [8]. A recent approach is to report longitudinal analyses of changes in consumption of foods and relate these to health outcomes. Examining associations for change in intake is valuable as eating behaviours change over time [9] and it is important to know if such change (either an increase or a decrease) can influence health outcomes. These data would provide public health officials an evidence base to give consumers clear advice regarding whether changing from being a low consumer to a high consumer, or vice versa, could have a real impact on health, even in mid-life. Moreover, as the data are derived within individuals, this approach is less prone to bias and confounding, and more closely resemble findings observed in randomised controlled trials [10].

The average intake of total flavonoids in the US (∼250 to ∼400 mg/day) is lower than the UK and Australia, and in line with intakes in Europe and China [5], with major dietary sources being tea, citrus fruit juices, berries, citrus fruit, wine, and apples [11]. While previous studies have demonstrated that higher intakes of specific flavonoid subclasses are associated with a lower risk of mortality [1, 4, 12], to our knowledge no studies have examined associations between changes in intakes of flavonoid-rich foods and risk of all-cause and cause-specific mortality. As foods are consumed as a whole, exploring associations for flavonoid-rich foods, as opposed to total flavonoids or flavonoid subclasses, yields results with a clearer interpretation and public health relevance. Flavonoids are found in a wide variety of plant-based foods and beverages, yet across the globe the major dietary sources of flavonoids tend to be very similar [13–16]; as such, deriving a composite score of flavonoid-rich foods is a novel and valuable method to better understanding the role of dietary flavonoids in disease prevention.

We therefore aimed to evaluate associations between 8-year changes in intakes of (1) individual flavonoid-rich foods and (2) a composite measure (termed the ‘flavodiet’) of foods and beverages that are known to be main contributors to flavonoid intake and subsequent total and cause-specific mortality among participants in the Nurses’ Health Study (NHS) and the Health Professionals Follow-up Study (HPFS). We hypothesised, on the basis of previous findings, that increases in intakes of some flavonoid-rich foods and beverages (namely, blueberry, apple, tea, and red wine) would be associated with a lower risk of all-cause mortality and that increases in the flavodiet score would be associated with a lower risk of cardiovascular disease-related, respiratory-related, and neurological disease-related mortality.

## Methods

### Study population

Data from two large prospective cohorts of US males and females were used for this study: the NHS began in 1976 and enrolled 121,701 female nurses aged 30–55 years [17] while the HPFS began in 1986 and enrolled 51,529 male health professionals aged 40–75 years [18]. In both cohorts, participants completed questionnaires about their lifestyle, and medical history at baseline and were invited to repeat these questionnaires every subsequent 2 years to collect and update information on lifestyle, and occurrence of new-onset diseases; follow-up rates for mortality exceed 98% for both cohorts.

The baseline for all analyses in the present study was set to be 1994, 8 years after detailed information on diet, physical activity, and other lifestyle factors had been collected for both cohorts, and the end of follow-up was 2018. We excluded participants who had prevalent cardiovascular disease, diabetes, cancer, or severe chronic neurological disease 4 years after baseline to reduce the likelihood of observed associations being due to reverse causality, participants with extreme energy intakes (< 800 or > 4200 kcal/d for men and < 600 or > 3500 kcal/d for women) which may reflect incorrect completion of the FFQ, and participants with missing information on change in flavonoid intakes and change in total fruit intakes. The final analysis included 55,786 females and 29,800 men (Additional file 1: Fig. S1). The last observation was carried forward for missing values of continuous variables (with the exception of diet) and indicators were used for missing values of categorical variables. Missing values were carried forward only once for diet, after which the follow-up was censored.

The study protocol was approved by the institutional review boards of the Brigham and Women’s Hospital and Harvard T.H. Chan School of Public Health, and those of participating registries as required.

### Dietary assessment

Participants of the two cohorts completed validated semi-quantitative food frequency questionnaires (FFQs) in 1986 and were invited to repeat these questionnaires every 4 years thereafter. Participants were asked how often, on average, they consumed a standard portion of each food and beverage in the past year with frequency response categories ranging from never or less than once a month, to six or more times per day. Exposures of interest were changes in intakes (servings/day) of foods and beverages that are known to be main contributors to flavonoid intake [namely, blueberry (½ cup), apple (1 fruit), orange (1 fruit) plus orange juice (1 small glass), grapefruit (½ fruit) plus grapefruit juice (1 small glass), strawberry (½ cup), tea (8 oz. cup), red wine (5 oz. glass), onion (1 slice raw or ½ cup cooked), peppers (2 rings or ¼ small), and grapes (½ cup) plus raisins (1 oz. or 1 small pack)] [16, 19]. Secondly, intakes of flavonoid-rich foods/beverages that contributed > 1% to total flavonoid intakes in both the NHS and the HPFS at all waves of follow-up were summed to create a ‘flavodiet’ score [i.e., tea, apples, oranges, grapefruits, blueberries, strawberries and red wine (servings/day)]. Of note, chocolate intake was not included as an exposure of interest as FFQs prior to 2006 did not assess intakes of milk and dark chocolate separately. The reproducibility and validity of the FFQs have been described previously [20, 21]. For the NHS, the corrected correlation coefficients, for several key flavonoid-rich foods, between the food frequency questionnaire and multiple dietary records were between 0.74 and 0.80 for apples, 0.50 and 0.74 for oranges, 0.86 and 0.93 for tea, and 0.83 and 0.90 for wine [22]. For the HPFS, these corrected correlation coefficients were between 0.44 and 0.53 for apples, 0.40 and 0.43 for oranges, 0.62 and 0.69 for tea, and 0.56 and 0.60 for red wine [23].

### Ascertainment of mortality

Death from any cause was the primary outcome for these analyses. Deaths were identified using the state vital statistics records, the national death index, reports by families, and the postal system; we ascertained over 98% of deaths in each cohort [24]. For all deaths, death certificates were sought and, when appropriate, permission was requested from the next of kin to review medical records. These death certificates and medical records were reviewed by a physician to determine the underlying cause of death according to the eighth and ninth revisions of the International Classification of Diseases (ICD) codes. Causes of death were grouped into five categories: cardiovascular disease-related, cancer-related, respiratory-related, neurological disease-related, and other). Due to heterogeneity in causes of death, the ‘other’ category, was not included it as an outcome of interest.

### Assessment of covariates

Participants reported their demographics (age, ethnicity, weight and height), lifestyle habits (physical activity, smoking habits, aspirin and multivitamin use), family history of diseases (myocardial infarction, diabetes and cancer) and any recent physician-diagnosed diseases (myocardial infarction, diabetes, cancer, hypertension, and hypercholesterolemia) via a questionnaire every 2 years. Physical activity was quantified as energy expenditure in metabolic equivalent tasks (METs) measured in hours per week as described previously [25]. As there was approximately 10% missing data for each question, response rates were carried over from the previous questionnaire. Dietary intakes of alcohol, total energy, meat, nuts, saturated fat, polyunsaturated fat, trans-fat, cereal fibre, and soft drink were assessed and updated from the food frequency questionnaire every 4 years.

### Statistical analysis

We calculated person-years of follow-up from the date of return of the 1994 questionnaire to the date of death or the end of follow-up, whichever came first. We used time-dependent Cox proportional hazards regression to estimate the hazard ratios (HRs) and 95% confidence intervals (CIs) of total and cause-specific mortality in the subsequent 6 years, after excluding the first 2 years of follow-up time in a lagged analysis; that is, changes in consumption between 1986 and 1994 predicted mortality between 1996 and 2002; changes in consumption between 1994 and 2002 predicted mortality between 2004 and 2010, and changes in consumption between 2002 and 2010 predicted mortality between 2012 and 2018. Proportional hazards assumptions were checked with no violations found. For the individual foods, participants were divided into seven categories based on their changes in consumption: three increase categories (increase of 0.5–0.99 servings/week; increase of 1–1.99 servings/week; increase of ≥ 2 servings/week); three decrease categories (decrease of 0.5–0.99 servings/week; decrease of 1–1.99 servings/week; decrease of ≥ 2 servings/week), and one reference category (no change; + / −  < 0.49 servings per week). Exposures were categorised as we did not wish to assume that associations were linear, a reference value of ‘no change’ was chosen so that different associations for an increase and a decrease in intake could be observed, and the category cut points were chosen based on the distribution of the data and ease of interpretation and translation. For the flavodiet score, participants were divided into seven categories based on their changes in consumption: three increase categories (increase of 1–3.9 servings/week; increase of 4–6.9 servings/week; increase of ≥ 7 servings/week); three decrease categories (decrease of 1–3.9 servings/week; decrease of 4–6.9 servings/week; decrease of ≥ 7 servings/week), and one reference category (increase or decrease of < 1 serving per week). Updated 8-year changes in consumption of individual flavonoid-rich foods, or the composite flavodiet score, were used as time-varying exposures. Eight years was chosen to allow enough time for people to change their diet and for that change to have an effect, as shown previously [26]. Risk of death (HR and 95% CI) was also estimated for a change in intake of each individual food or beverage by 3.5 servings/week, representing a serving every second day, except for tea intake which was estimated for 7 servings/week, representing a serving every day. For the flavodiet score, the risk of death (HR and 95% CI) was also estimated for a change total intake of 3 servings/day. We calculated HRs and 95% CIs from the different models separately for each cohort and then pooled the results of Model 2 from both cohorts using a fixed-effects meta-analysis. As the between-study variance heterogeneity tests were not significant for most of the main exposures, a fixed-effects meta-analysis was used.

Model 1 adjusted for baseline age (calendar year) and baseline intake of the exposure variable of interest while Model 2 adjusted for baseline age, follow-up time periods, ethnicity (white v other), change in smoking status (never to never, never to current, former to former, former to current, current to former, current to current, or missing indicator), a family history of myocardial infarction, diabetes and cancer (all yes v no), multivitamin use (yes v no), aspirin use (yes v no), history of hypertension, hypercholesterolemia, and diabetes (all yes v no), baseline physical activity (quintiles), change in physical activity, baseline BMI (< 23, 23–24.9, 25–29.9, 30–34.9, > 35 kg/m^2^), change in BMI, baseline intakes of the exposure variable of interest, and both baseline and change in intakes of alcohol, total energy, meat, nuts, saturated fat, polyunsaturated fat, trans fat, cereal fibre, and soft drink (all servings/day).

To investigate potential effect modification of the association between change in the flavodiet score and mortality (all-cause and cause-specific), we stratified our analyses by BMI (< 30 v ≥ 30 kg/m^2^) and smoking status (never v ever) based on prior evidence of effect modification [1]. Furthermore, we conducted sensitivity analyses where we censored participants at 80 years of age so that our outcome would represent premature death. All analyses used SAS version 9.2 (SAS Institute, Cary, NC) and statistical tests were two-sided and a *P* value < 0.05 was considered statistically significant.

## Results

We documented 15,293 deaths (including 3401 CVD deaths, 3530 cancer deaths, 1282 respiratory deaths and 2475 neurological deaths) in the NHS over 843,190 person-years of follow-up and 8988 deaths (including 2634 CVD deaths, 2345 cancer deaths, 677 respiratory deaths and 828 neurological deaths) in HPFS over 423,565 person-years of follow-up. Table 1 shows the characteristics of the participants based on an 8-year change in the flavodiet score from 1986 to 1994. From 1986 to 1994, more participants increased their intakes of flavonoid-rich foods than decreased their intakes in both cohorts (Table 1). Although body weight tended to increase over follow-up for all participants, those with the greatest increase in flavonoid-rich food intake appeared to be healthier in that they had, on average, a lower increase in body weight and a greater increase in both energy intake and physical activity than participants who decreased their flavonoid-rich food intake. Participants who maintained a similar level of flavonoid-rich food intake were more likely to be current smokers than those who increased or decreased their intakes. Mean intakes of most flavonoid-rich foods remained stable over time (1986 to 2010) except for intakes of blueberries, tea, and red wine, which tended to increase in both cohorts, and intakes of citrus fruits (orange and grapefruit) and their juices which tended to decrease (Additional file 1: Fig. S2).

**Table 1:** Age-adjusted characteristics of participants based on 8-year changes (1986–1994) in the flavodiet score (servings/week)

	Decrease in flavodiet score (servings/week)				Increase in flavodiet score (servings/week)		
Characteristics	≥ 7	4–6.9	1–3.9	Reference (< 1)	1–3.9	4–6.9	≥ 7
***Nurses’ Health Study***							
No. of participants	6,986	4,511	8,655	11,567	9,830	5,458	8,779
Age (years)	60.3 (7.0)	60.4 (7.0)	60.1 (7.1)	59.9 (7.1)	60.2 (7.1)	60.1 (7.1)	60.2 (7.1)
Initial alcohol intake (g/day)	6.4 (11.2)	6.8 (11.2)	6.3 (10.5)	6.5 (11.3)	6.0 (10.2)	5.7 (9.7)	5.6 (9.8)
Change in alcohol intake (g/day)	− 1.7 (8.1)	− 1.5 (8.5)	− 1.3 (7.6)	− 1.4 (7.9)	− 1.0 (7.6)	− 0.9 (7.1)	− 0.6 (8.0)
Initial physical activity (MET h/week)	15.3 (22.3)	15.4 (20.7)	14.4 (20.3)	12.8 (18.5)	13.5 (17.7)	14.9 (22.2)	14.6 (21.0)
Change in physical activity (MET h/week)	4.4 (27.4)	4.3 (26.2)	4.9 (25.3)	5.0 (23.0)	6.3 (22.9)	6.8 (26.3)	7.5 (28.6)
Initial energy intake (calories/day)	1869.9 (544.7)	1834.7 (522.1)	1789.2 (513.9)	1709.6 (508.0)	1735.0 (507.5)	1740.5 (513.1)	1752.1 (506.8)
Change in energy intake (calories/d)	− 156.9 (493.3)	− 121.2 (486)	− 90.5 (463.4)	− 42.6 (455.3)	16.5 (457.9)	58.0 (468.0)	88.9 (496.2)
Initial body mass index (kg/m^2^)	25.4 (4.7)	25.4 (4.9)	25.4 (4.7)	25.2 (4.6)	25.0 (4.5)	25.1 (4.7)	25.2 (4.7)
Change in body mass index (kg/m^2^)	1.2 (2.5)	1.2 (2.4)	1.1 (2.4)	1.1 (2.3)	1.1 (2.3)	1.0 (2.4)	1.0 (2.4)
Initial weight (kg)	68.3 (13.5)	68.1 (13.7)	68.2 (13.6)	67.6 (13.2)	67.3 (12.8)	67.5 (13.2)	67.6 (13.4)
Weight change (kg)	3.3 (6.7)	3.3 (6.5)	3.0 (6.4)	3.0 (6.3)	3.0 (6.2)	2.7 (6.3)	2.8 (6.5)
Parental history of MI (%)^a^	25.5	23.2	24.3	23.4	23.8	24.1	24.8
Hypertension (%)^a^	37.7	36.3	35.9	36.2	35.7	35.6	37.4
High cholesterol level (%)^a^	49.8	48.8	49.1	49.5	49.2	51.1	49.7
Type 2 diabetes (%)^a^	6.0	5.5	5.3	5.2	5.5	5.8	6.5
Current smoker (%)^a^	18.7	16.5	19.1	23.5	19.3	18.5	18.3
Change in smoking status (%)							
Current to past	7.6	6.5	7.2	8.3	8.3	8.8	8.2
Past to current	0.6	0.9	0.8	0.8	0.7	0.6	0.7
Never to current	0.0	0.0	0.0	0.0	0.0	0.0	0.0
Multivitamin (%)^a^	47.9	47.2	46.5	45.2	46.9	48.7	49.0
Aspirin (%)^a^	43.5	43.9	44.1	44.6	44.1	43.7	44.4
***Health Professionals Follow-Up Study***							
No. of participants	3,447	2,354	4,901	6,171	5,493	2,860	4,574
Age (years)	61.4 (9.2)	61.3 (9.3)	60.8 (9.2)	60.2 (9.2)	60.8 (9.1)	61.5 (9.3)	61.3 (9.3)
Initial alcohol intake (g/day)	11.6 (16.3)	11.2 (15.3)	11.5 (14.7)	11.6 (15.3)	11.4 (14.6)	11.0 (14.1)	11.7 (15.5)
Change in alcohol intake (g/day)	− 1.4 (12.7)	− 1.1 (11.2)	− 0.6 (11.2)	− 0.3 (11.0)	0 (10.8)	0.3 (11.1)	0.9 (12.6)
Initial physical activity (MET h/week)	20.7 (29.0)	20.7 (26.6)	20.3 (30.8)	17.3 (24.0)	18.5 (23.6)	19.4 (25.7)	19.5 (24.6)
Change in physical activity (MET h/week)	10.6 (33.7)	10.4 (30.0)	10.4 (33.4)	11.3 (28.7)	13.0 (29.4)	13.3 (31.1)	13.9 (30.4)
Initial energy intake (calories/day)	2133.6 (622.2)	2034.1 (584.6)	2023.9 (604.0)	1934.8 (568.6)	1946.0 (580.6)	1966.6 (582.3)	1975.2 (586.9)
Change in energy intake (calories/day)	− 148.3 (569.2)	− 92.9 (519.5)	− 49.3 (532.8)	10.6 (524.3)	71.0 (525.3)	97.6 (548.1)	181.9 (560.0)
Initial body mass index (kg/m^2^)	25.4 (3.3)	25.4 (3.0)	25.3 (3.0)	25.3 (3.1)	25.3 (3.0)	25.3 (3.0)	25.5 (3.2)
Change in body mass index (kg/m^2^)	0.6 (1.8)	0.6 (1.6)	0.6 (1.5)	0.5 (1.5)	0.5 (1.5)	0.4 (1.4)	0.4 (1.6)
Initial weight (kg)	81.0 (12.0)	80.8 (11.1)	80.6 (11.1)	80.6 (11.3)	80.8 (11.0)	80.7 (11.0)	81.2 (11.9)
Weight change (kg)	2.0 (5.7)	1.9 (5.0)	1.8 (4.9)	1.6 (4.7)	1.5 (4.8)	1.4 (4.5)	1.4 (5.3)
Parental history of MI (%)^a^	38.5	36.9	36.2	36.8	37.4	37.6	37.6
Hypertension (%)^a^	32.8	29.9	30.3	29.3	30.6	30.1	31.6
High cholesterol level (%)^a^	40.6	40.2	39.4	39.9	42.0	41.0	40.8
Type 2 diabetes (%)^a^	5.1	5.0	4.1	3.9	4.6	4.7	4.8
Current smoker (%)^a^	7.4	7.4	7.3	10.9	8.4	7.3	7.8
Change in smoking status (%)							
Current to past	3.4	3.5	2.7	4.1	4.0	4.3	4.2
Past to current	0.7	1.3	1.1	1.3	1.0	0.8	0.8
Never to current	0.1	0.0	0.1	0.1	0.1	0.1	0.0
Multivitamin (%)^a^	50.2	50.6	49.8	46.9	47.9	49.7	49.7
Aspirin (%)^a^	60.0	59.1	57.8	56.9	59.2	58.4	61.1

*MET* metabolic equivalent of task, *HRT* hormone replacement therapy, *MI* myocardial infarction

Values are standardised to the age distribution of the study population (except age). Continuous variables are expressed as mean (standard deviation) while binary variables are expressed as %. ^a^Represents participant status in 1986

Age-adjusted (Model 1) associations between changes in intakes of flavonoid-rich foods and total mortality are presented in Additional file 1: Table S1. In pooled multivariable-adjusted analyses, compared with participants whose intakes remained relatively stable, those with the greatest increase in intakes of tea, red wine and peppers had a 5%, 11%, and 6% lower risk of total mortality, respectively [pooled HR (95% CI) for tea: 0.95 (0.92, 0.98); red wine: 0.89 (0.84, 0.95); and peppers: 0.94 (0.90, 0.98); Table 2]. When modelled as a continuous variable, a 1 serving/day increase in tea intake was associated with a 3% lower all-cause mortality while 1 serving every other day (3.5 servings/week) increases in intakes of red wine, peppers and blueberries were associated with 4%, 9% and 5% lower risks of all-cause mortality in multivariable-adjusted pooled analyses, respectively. For blueberries, this appeared to be driven by a trend for a high risk of mortality in participants who decreased their intakes of blueberries. In contrast, those with the greatest increase in intakes of strawberries, onions and citrus fruits and their juices had an 8% (strawberry), 6% (onion), 8% (orange) and 6% (grapefruit) higher risk of mortality after multivariable adjustments (Table 2). When modelled as a continuous variable, a 1 serving every other day (3.5 servings/week) increase in intakes of grapefruit and onion was associated with a 6% and 5% higher risks of all-cause mortality in multivariable-adjusted pooled analyses, respectively.

**Table 2:** Associations between all-cause mortality (hazard ratios, 95% confidence intervals)^a^ and 8-year change in intake of flavonoid-rich foods

	8-year change in intake levels, servings/week								
	Decrease				Increase				
	≥ 2	1–1.99	0.5–0.99	No change (± 0.49)	0.5–0.99	1–1.99	≥ 2	*P*_trend_	Every 3.5 servings/week change^e^
***Blueberry (fresh, frozen or canned)***									
NHS^b^		529/22,972	398/17,844	12,448/702,562	579/32,856	1339/66,349			
Model 2^c^		1.21 (1.06, 1.37)	1.06 (0.96, 1.18)	1.00	0.95 (0.87, 1.04)	0.96 (0.91, 1.02)			0.90 (0.85, 0.95)
HPFS^b^		202/7729	111/4209	7503/367,546	280/12,191	892/31,890			
Model 2^c^		1.06 (0.89, 1.27)	1.14 (0.93, 1.38)	1.00	0.98 (0.87, 1.11)	1.02 (0.95, 1.10)			1.01 (0.95, 1.07)
**Pooled**^**d**^		**1.16 (1.04, 1.28)**	**1.08 (0.98, 1.18)**	**1.00**	**0.96 (0.89, 1.03)**	**0.98 (0.94, 1.03)**		**0.01**	**0.95 (0.91, 0.99)**
***Apple (fresh)***									
NHS^b^	3196/175,003	110/7270	440/16,291	8798/475,020	234/10,834	91/6427	2424/151,739		
Model 2^c^	1.04 (0.99, 1.10)	1.08 (0.88, 1.32)	1.06 (0.96, 1.17)	1.00	0.98 (0.86, 1.12)	1.03 (0.83, 1.28)	1.04 (0.99, 1.09)		1.00 (0.97, 1.03)
HPFS^b^	2093/89,216	125/5321	139/4822	4762/231,582	99/4407	101/5351	1669/82,867		
Model 2^c^	1.04 (0.97, 1.10)	1.10 (0.91, 1.33)	1.10 (0.92, 1.31)	1.00	1.07 (0.87, 1.31)	0.93 (0.75, 1.14)	1.01 (0.95, 1.07)		0.97 (0.94, 1.00)
**Pooled**^**d**^	**1.04 (1.00, 1.08)**	**1.09 (0.95, 1.25)**	**1.07 (0.98, 1.17)**	**1.00**	**1.01 (0.90, 1.13)**	**0.98 (0.84, 1.13)**	**1.03 (0.99, 1.07)**	**0.17**	**0.98 (0.96, 1.01)**
***Orange and orange juice***									
NHS^b^	4349/229,783	654/34,599	683/40,348	5304/297,549	511/26,651	513/29,248	3279/184,405		
Model 2^c^	1.04 (0.99, 1.09)	1.00 (0.92, 1.09)	0.92 (0.85, 1.00)	1.00	1.11 (1.02, 1.22)	1.08 (0.98, 1.18)	1.10 (1.05, 1.15)		1.02 (1.00, 1.04)
HPFS^b^	2613/114,822	355/16,461	329/16,253	2789/138,532	255/13,691	325/16,196	2322/107,611		
Model 2^c^	1.00 (0.94, 1.06)	0.90 (0.80, 1.01)	1.01 (0.90, 1.13)	1.00	0.90 (0.79, 1.03)	0.91 (0.81, 1.02)	1.05 (0.99, 1.11)		1.00 (0.99, 1.02)
**Pooled**^**d**^	**1.02 (0.99, 1.06)**	**0.96 (0.9, 1.03)**	**0.95 (0.89, 1.01)**	**1.00**	**1.05 (0.98, 1.14)**	**1.01 (0.94, 1.09)**	**1.08 (1.05, 1.12)**	**0.03**	**1.01 (1.00, 1.03)**
***Tea***									^e^1 serving/day
NHS^b^	3495/177,247	255/12,515	484/21,256	7288/397,330	417/26,195	184/11,547	3,170/196,493		
Model 2^c^	1.02 (0.97, 1.07)	0.94 (0.82, 1.07)	1.11 (1.01, 1.22)	1.00	0.93 (0.84, 1.03)	0.85 (0.73, 0.99)	0.93 (0.89, 0.97)		0.96 (0.95, 0.98)
HPFS^b^	1726/75,078	132/5679	269/11,771	4892/233,143	273/13,577	126/6,160	1,570/78,156		
Model 2^c^	1.05 (0.98, 1.12)	0.88 (0.73, 1.05)	1.07 (0.94, 1.21)	1.00	0.99 (0.87, 1.12)	0.90 (0.75, 1.08)	0.99 (0.93, 1.05)		0.98 (0.95, 1.00)
**Pooled**^**d**^	**1.03 (0.99, 1.07)**	**0.94 (0.85, 1.04)**	**1.10 (1.02, 1.18)**	**1.00**	**0.95 (0.88, 1.03)**	**0.87 (0.78, 0.98)**	**0.95 (0.92, 0.98)**	**< 0.0001**	**0.97 (0.95, 0.98)**
***Grapefruit and grapefruit juice***									
NHS^b^	1964/118,026	253/20,041	1114/62,321	10,762/547,361	280/20,955	114/9472	806/64,407		
Model 2^c^	0.95 (0.88, 1.01)	0.94 (0.82, 1.06)	1.03 (0.97, 1.10)	1.00	1.18 (1.05, 1.34)	1.19 (0.99, 1.43)	1.04 (0.97, 1.13)		1.06 (1.02, 1.10)
HPFS^b^	1488/57,102	131/7765	505/24,566	5617/272,260	210/12,091	99/5824	938/43,958		
Model 2^c^	1.00 (0.92, 1.08)	0.96 (0.80, 1.15)	1.03 (0.94, 1.14)	1.00	1.19 (1.03, 1.38)	0.97 (0.79, 1.19)	1.08 (1.00, 1.16)		1.04 (1.00, 1.09)
**Pooled**^**d**^	**0.97 (0.92, 1.02)**	**0.94 (0.84, 1.04)**	**1.03 (0.98, 1.09)**	**1.00**	**1.19 (1.08, 1.30)**	**1.10 (0.96, 1.27)**	**1.06 (1.01, 1.12)**	**0.0002**	**1.06 (1.03, 1.09)**
***Red wine***									
NHS^b^		672/37,113	219/13,187	13,481/717,922	173/15,856	748/58,505			
Model 2^c^		1.00 (0.90, 1.11)	0.92 (0.80, 1.05)	1.00	0.84 (0.72, 0.99)	0.88 (0.81, 0.96)			0.96 (0.91, 1.00)
HPFS^b^		796/30,425	179/6,864	6889/313,529	172/11,094	952/61,654			
Model 2^c^		1.04 (0.95, 1.15)	1.15 (0.99, 1.34)	1.00	0.88 (0.75, 1.03)	0.91 (0.84, 0.98)			0.96 (0.93, 1.00)
**Pooled**^d^		**1.02 (0.95, 1.10)**	**1.01 (0.91, 1.12)**	**1.00**	**0.86 (0.77, 0.96)**	**0.89 (0.84, 0.95)**		**0.006**	**0.96 (0.93, 0.99)**
***Strawberry (fresh, frozen or canned)***									
NHS^b^		1148/58,820	642/23,736	11,607/657,567	423/21,245	1473/81,215			
Model 2^c^		1.09 (1.00, 1.18)	1.12 (1.04, 1.22)	1.00	1.02 (0.92, 1.12)	1.05 (1.00, 1.12)			0.99 (0.94, 1.04)
HPFS^b^		402/16,312	201/7,525	7226/358,344	239/10,439	920/30,946			
Model 2^c^		0.93 (0.81, 1.06)	1.11 (0.96, 1.29)	1.00	1.01 (0.88, 1.15)	1.11 (1.03, 1.20)			1.09 (1.02, 1.16)
**Pooled**^**d**^		**1.04 (0.97, 1.12)**	**1.12 (1.04, 1.20)**	**1.00**	**1.01 (0.94, 1.10)**	**1.07 (1.03, 1.12)**		**0.19**	**1.03 (0.99, 1.07)**
***Onion (raw or cooked)***									
NHS^b^		1766/99,802	1063/56,193	11,405/612,615	248/15,510	811/58,464			
Model 2^c^		1.00 (0.95, 1.05)	1.00 (0.93, 1.06)	1.00	1.13 (1.00, 1.28)	1.08 (1.00, 1.16)			1.05 (1.00, 1.09)
HPFS^b^		968/36,949	236/11,251	6123/306,015	340/17,304	1321/52,047			
Model 2^c^		0.98 (0.89, 1.08)	0.93 (0.81, 1.06)	1.00	0.95 (0.85, 1.06)	1.04 (0.98, 1.11)			1.05 (0.99, 1.10)
**Pooled**^**d**^		**0.99 (0.94, 1.04)**	**0.98 (0.92, 1.04)**	**1.00**	**1.03 (0.95, 1.12)**	**1.06 (1.01, 1.11)**		**0.007**	**1.05 (1.01, 1.08)**
***Peppers***									
NHS^b^		2239/117,437	579/20,356	10,764/575,134	265/16,902	1446/112,754			
Model 2^c^		1.14 (1.07, 1.22)	1.21 (1.12, 1.32)	1.00	1.00 (0.88, 1.13)	0.93 (0.88, 0.98)			0.88 (0.84, 0.92)
HPFS^b^		1177/46,636	189/7133	6295/299,699	184/10,711	1143/59,416			
Model 2^c^		1.03 (0.95, 1.12)	0.97 (0.83, 1.12)	1.00	1.06 (0.91, 1.24)	0.96 (0.90, 1.03)			0.97 (0.92, 1.02)
**Pooled**^**d**^		**1.10 (1.05, 1.16)**	**1.15 (1.07, 1.24)**	**1.00**	**1.02 (0.93, 1.12)**	**0.94 (0.90, 0.98)**		**< 0.0001**	**0.91 (0.88, 0.95)**
***Grape and raisin***									
NHS^b^		1576/79,843	593/30,650	10,839/599,807	645/36,896	1640/95,387			
Model 2^c^		1.00 (0.95, 1.06)	0.96 (0.89, 1.04)	1.00	0.95 (0.88, 1.03)	0.93 (0.88, 0.98)			0.95 (0.92, 0.99)
HPFS^b^		1,185/45,439	236/10,302	5923/299,458	253/11,719	1391/56,648			
Model 2^c^		1.05 (0.97, 1.13)	0.99 (0.87, 1.13)	1.00	1.12 (0.98, 1.27)	1.05 (0.99, 1.12)			1.00 (0.96, 1.04)
**Pooled**^**d**^		**1.02 (0.97, 1.07)**	**0.98 (0.91, 1.05)**	**1.00**	**1.00 (0.93, 1.07)**	**0.98 (0.94, 1.02)**		**0.048**	**0.97 (0.95, 1.00)**

^a^All hazard ratios (95% confidence intervals) were calculated using Cox proportional hazard models. *HPFS*, Health Professionals Follow-up Study; *NHS*, Nurses’ Health Study

^b^Cases/person-years

^c^Model 2 was adjusted for age, time periods, ethnicity, change in smoking status, a family history of myocardial infarction, diabetes and cancer, multivitamin use, aspirin use, history of hypertension, hypercholesterolemia, and diabetes, baseline physical activity, change in physical activity, baseline BMI, change in BMI, baseline intakes of the exposure variable, and both baseline and change in intakes of alcohol, total energy, meat, nuts, saturated fat, polyunsaturated fat, trans-fat, cereal fibre, and soft drink

^d^Results of Model 2 from both cohorts were pooled using a fixed-effects meta-analysis

^e^Except for tea, where the hazard ratio (95% CI) is presented for an 8-year change in intake of 1 serve per day

**Fig. 1:**
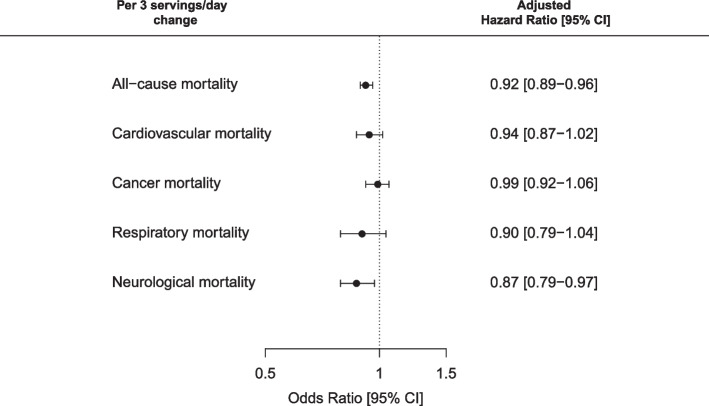
Associations between 8-year change in flavodiet score and both all-cause and cause-specific mortality. Hazard ratios and 95% confidence intervals were obtained from a pooled analysis (fixed-effects meta-analysis) of the Nurses’ Health Study and the Health Professionals Follow-up Study and are presented for an 8-year change in intakes of + 3 servings/day. Analyses are adjusted for age, time periods, ethnicity, change in smoking status, a family history of myocardial infarction, diabetes and cancer, multivitamin use, aspirin use, history of hypertension, hypercholesterolemia, and diabetes, baseline physical activity, change in physical activity, baseline BMI, change in BMI, baseline intakes of each flavonoid-rich food combined to create the flavodiet score, and both baseline and change in intakes of alcohol, total energy, meat, nuts, saturated fat, polyunsaturated fat, trans-fat, cereal fibre, and soft drink

When intakes of the top contributors to total flavonoid intake were combined to create a flavodiet score, a 3 servings/day increase in this combined score was associated with an 8% lower risk of all-cause mortality, after multivariable adjustments [pooled HR (95% CI): 0.92 (0.89, 0.96); Fig. 1]. Participants who had the greatest decrease in flavodiet score (≥ 7 servings/week) had an 11% higher risk of mortality [pooled HR (95% CI): 1.11 (1.05, 1.17)] than those whose score remained stable (Table 3). Although an increase in flavodiet score was not significantly associated with a reduction in risk of cardiovascular, cancer or respiratory mortality, an increase of 3 servings per day of the flavodiet score was associated with a 13% reduction in risk of neurological death (Fig. 1) and participants who had the greatest decrease in flavodiet score (≥ 7 servings/week) had a 14% higher risk of neurological mortality [pooled HR (95% CI): 1.14 (1.00, 1.31)] than those whose score remained stable (Table 3).

**Table 3:** Associations between all-cause and cause-specific mortality (hazard ratios, 95% confidence intervals)^a^ and 8-year change in flavodiet score

	8-year change in intake levels, serving/wk							
		Decrease				Increase		
	7.0 or more	4.0–6.9	1.0–3.9	No change (± 0.9)	1.0–3.9	4.0–6.9	7.0 or more	*P*_trend_
***All-cause mortality***								
NHS								
Model 2^b^	1.10 (1.03, 1.17)	1.01 (0.94, 1.08)	0.96 (0.91, 1.01)	1.00	0.95 (0.90, 1.01)	0.97 (0.91, 1.03)	0.95 (0.90, 1.01)	
HPFS								
Model 2^b^	1.11 (1.02, 1.21)	1.00 (0.91, 1.09)	0.90 (0.83, 0.97)	1.00	0.99 (0.92, 1.06)	0.99 (0.91, 1.07)	0.98 (0.91, 1.06)	
**Pooled**^**c**^	**1.10 (1.05, 1.16)**	**1.00 (0.95, 1.06)**	**0.94 (0.90, 0.98)**	**1.00**	**0.97 (0.93, 1.01)**	**0.97 (0.92, 1.02)**	**0.96 (0.92, 1.01)**	**< 0.0001**
***Cardiovascular mortality***								
NHS								
Model 2^b^	1.09 (0.95, 1.24)	0.98 (0.85, 1.12)	1.02 (0.91, 1.14)	1.00	0.91 (0.80, 1.02)	0.96 (0.84, 1.10)	0.94 (0.84, 1.07)	
HPFS								
Model 2^b^	1.13 (0.96, 1.32)	1.09 (0.92, 1.28)	0.93 (0.81, 1.06)	1.00	1.00 (0.87, 1.14)	1.12 (0.97, 1.30)	1.01 (0.88, 1.16)	
**Pooled**^**c**^	**1.10 (0.99, 1.22)**	**1.02 (0.92, 1.14)**	**0.98 (0.90, 1.07)**	**1.00**	**0.94 (0.86, 1.03)**	**1.03 (0.93, 1.14)**	**0.97 (0.89, 1.07)**	**0.11**
***Cancer mortality***								
NHS								
Model 2^b^	0.99 (0.87, 1.14)	1.03 (0.90, 1.18)	0.94 (0.84, 1.05)	1.00	1.01 (0.91, 1.13)	0.96 (0.84, 1.10)	0.97 (0.86, 1.09)	
HPFS								
Model 2^b^	1.01 (0.85, 1.19)	0.85 (0.71, 1.01)	0.78 (0.67, 0.90)	1.00	0.95 (0.83, 1.08)	0.94 (0.80, 1.09)	0.92 (0.80, 1.05)	
**Pooled**^**c**^	**1.00 (0.90, 1.11)**	**0.96 (0.86, 1.07)**	**0.87 (0.80, 0.95)**	**1.00**	**0.98 (0.90, 1.07)**	**0.95 (0.86, 1.05)**	**0.95 (0.87, 1.03)**	**0.73**
***Respiratory mortality***								
NHS								
Model 2^b^	1.15 (0.93, 1.43)	1.05 (0.84, 1.31)	0.87 (0.72, 1.04)	1.00	0.90 (0.76, 1.08)	1.18 (0.96, 1.45)	1.01 (0.83, 1.22)	
HPFS								
Model 2^b^	1.30 (0.94, 1.78)	0.94 (0.67, 1.32)	1.03 (0.79, 1.34)	1.00	0.97 (0.74, 1.27)	0.92 (0.66, 1.26)	1.00 (0.76, 1.31)	
**Pooled**^**c**^	**1.20 (1.00, 1.44)**	**1.01 (0.84, 1.22)**	**0.92 (0.79, 1.07)**	**1.00**	**0.92 (0.79, 1.08)**	**1.09 (0.92, 1.30)**	**1.00 (0.86, 1.18)**	**0.15**
***Neurological mortality***								
NHS								
Model 2^b^	1.09 (0.94, 1.28)	0.93 (0.79, 1.07)	0.98 (0.86, 1.12)	1.00	0.93 (0.81, 1.06)	0.91 (0.77, 1.07)	0.98 (0.85, 1.13)	
HPFS								
Model 2^b^	1.33 (1.01, 1.77)	0.97 (0.72, 1.31)	0.90 (0.70, 1.15)	1.00	1.09 (0.86, 1.37)	0.84 (0.63, 1.12)	0.92 (0.71, 1.18)	
**Pooled**^**c**^	**1.14 (1.00, 1.31)**	**0.94 (0.81, 1.08)**	**0.96 (0.86, 1.08)**	**1.00**	**0.97 (0.86, 1.09)**	**0.89 (0.78, 1.03)**	**0.97 (0.85, 1.09)**	**0.012**

^a^All hazard ratios (95% confidence intervals) were calculated using Cox proportional hazard model. *HPFS*, Health Professionals Follow-up Study; *NHS*, Nurses’ Health Study

^b^Model 2 was adjusted for age, time periods, ethnicity, change in smoking status, a family history of myocardial infarction, diabetes and cancer, multivitamin use, aspirin use, history of hypertension, hypercholesterolemia, and diabetes, baseline physical activity, change in physical activity, baseline BMI, change in BMI, baseline intakes of each flavonoid-rich food combined to create the flavodiet score, and both baseline and change in intakes of alcohol, total energy, meat, nuts, saturated fat, polyunsaturated fat, trans-fat, cereal fibre, and soft drink

^c^Results of model 2 from both cohorts were pooled using a fixed-effects meta-analysis

In sensitivity analyses, censoring participants at 80 years did not materially change the results. Furthermore, we found no evidence of effect modification by smoking status (ever v never) or BMI when examining associations between the flavodiet score and either all-cause or cause-specific mortality in each cohort (*p*_interaction_ > 0.1 for all).

## Discussion

In this prospective cohort study of well-characterised US females and males with repeated measures of dietary intake to allow for the calculation of an 8-year change in consumption of flavonoid-rich foods and beverages, we observed that an achievable increase in intake of several flavonoid-rich foods, namely tea, blueberries, red wine and peppers, was associated with up to an 11% reduction in all-cause mortality. Furthermore, by deriving a novel flavodiet score we showed that an increase of three servings per day of any of the main contributors to total flavonoid intake was associated with an 8% and 13% lower risk of all-cause and neurological death, respectively. These novel findings highlight that modifying eating behaviours in mid to later life can influence health outcomes and that several flavonoid-rich foods may offer distinct benefits.

To date, most observational studies aiming to understand the role of dietary flavonoids in the prevention of chronic disease and subsequent early mortality have focussed on total flavonoid intakes and intakes of major flavonoid subclasses and compounds [5]. However, the overall health effect may depend on the other components that they are consumed alongside as part of the whole food matrix [27]. Thus, there is also a need to evaluate flavonoids in the context of the whole food; one way in which this can be done is by examining associations between intakes of flavonoid-rich foods and health outcomes. In the present study, increased intakes of not all flavonoid-rich foods were associated with a lower risk of total mortality. Of the fruits, decreasing intakes of blueberries and increasing intakes of strawberries and citrus fruits and their juices were associated with a higher risk of total mortality. In the case of strawberries, decreasing intakes were also associated with a higher risk of mortality and for both strawberries and oranges the test for trend was not statistically significant; thus, findings are less convincing and may be due to chance, particularly given the large number of statistical tests undertaken. The differential associations for blueberries and citrus, grapefruit in particular, may be explained by other, non-beneficial, properties of the citrus fruits and juices, namely the high glycaemic load of juice [28] and the known interaction between grapefruit and drug metabolism [29]. Regarding flavonoid-rich vegetables, a 3.5 servings per week increase in onion intake was associated with a 5% higher risk of mortality while the same increase in intakes of peppers was associated with a 9% lower risk of all-cause mortality. As onions and peppers are rarely eaten in isolation, their health effects are inherently difficult to disentangle from effects of the whole meal in an observational setting and the observed associations should be interpreted with caution. Associations observed in the present study are comparable to those reported previously for the NHS II cohort of younger females, where frequent consumers of blueberries, peppers, red wine and tea had a lower risk of mortality while grapefruit consumers were at a higher risk [4]. Conversely, no association was observed between onion intakes and mortality in the NHS II. Plant-derived beverages are also an important dietary source of bioactive compounds including flavonoids. In our pooled analyses, increases in tea and red wine intakes were inversely associated with all-cause mortality risk, in line with findings from previous meta-analyses [30, 31]. Based on findings from both epidemiological and interventional studies, a small to moderate intake of red wine daily is thought to be cardioprotective, with evidence of a synergistic effect between the polyphenols and the ethanol found in red wine [32]. However, alcohol is also a major risk factor for global disease and economic burden [33]. Taken together, findings from the present study suggest that increasing intakes of particular flavonoid-rich foods, even in mid-life, may possibly have a real impact on risk of early mortality.

For the flavodiet score, we showed that an achievable three servings/day increase in intake of flavonoid-rich foods (for example one cup of tea, one serving of blueberries and one glass of red wine) was associated with an 8% lower risk of all-cause mortality in our multivariable-adjusted pooled analysis. This appeared to be driven by an increase in risk seen for participants with the greatest decreases in flavodiet score. To date, a wealth of evidence suggests that increased intakes of flavonoid-rich foods, including tea and berries, and several sub-classes of flavonoids, are inversely associated with biomarkers and risk of cardiovascular disease [3, 5, 30]. The most commonly investigated role for flavonoids in in vitro and animal studies relates to their potential cardioprotective role including regulation of nitric oxide synthase, COX-2 expression, and platelet aggregation [6]. The lack of association between an increase in the flavodiet score and cardiovascular mortality was therefore surprising but may be explained by the diversity of foods, and their flavonoid composition, within the flavodiet score, not all of which may be cardioprotective. As well as for cardiovascular disease, chronic inflammation plays a key role in respiratory and neurodegenerative disease [34] and many flavonoid metabolites have been shown to interact with chronic inflammatory disease at a molecular level and modulate the response of key enzymes and cell-signalling cascades [6, 34, 35]. In population-based studies, a higher habitual intake of several flavonoids, including anthocyanins and flavonols, was associated with a lower level of inflammation [36, 37]. This may explain the observation in the present study that an increase of three servings/day of the flavodiet score was associated with a 13% lower risk of mortality from neurological causes and a 10% lower risk of respiratory mortality, although the latter did not reach statistical significance. As for all-cause mortality, this appeared to be driven by the higher risk of mortality from neurological causes seen for participants with the greatest decreases in flavodiet score. The limited data in human studies suggest that higher habitual flavonoid intakes are positively associated with lung function [38, 39] and we have recently shown that higher flavonoid intakes are inversely associated with incident COPD in current and former, but not in never, smokers [40]. While we observed that a decrease in flavodiet score was associated with an increased risk of mortality in the present study, we observed no interaction with smoking status. There is more evidence for a protective role of flavonoids in the development of neurological diseases [41] with mechanisms including a reduction in reactive oxygen species and amyloid beta-protein production [42]. Recent data also suggest that higher intake of flavonoids and flavonoid-rich foods are associated with a lower risk of Alzheimer’s, dementia, and Parkinson’s disease [43–46] but our data are the first to suggest that increasing intakes in mid-life can reduce risk of neurological death. Although we include a lag time of 2 years, we cannot discount the possibility that observed associations may be attributed to reverse causation, in particular, that a higher risk of neurological death may be linked to a decrease in intakes of flavonoid-rich foods. This is plausible because changes in diet due to neurologic degenerative diseases may occur many years before death and previous research reports that individuals with Alzheimer’s disease and dementia, which make up a large proportion of neurological deaths, have accelerated weight loss [47], malnutrition [48], and food preferences for sweet and salty foods [49]. However, we also report a lower risk of neurological death with an increase in intakes of flavonoid-rich foods, a finding less susceptible to reverse causation when the comparator is those whose intakes did not change.

Strengths of this study include the prospective design, large sample size with long-term follow-up, repeated measures of dietary intake which allowed us to calculate the updated 8-year change in intakes, and detailed data on important risk factors and confounders. A potential limitation of the flavodiet score is the variability in the flavonoid composition between the seven foods and beverages. However, our analyses focus on flavonoid-rich foods and a flavodiet score, rather than specific flavonoid sub-classes and a weighted score, so that the public health relevance of the findings can be fully appreciated. While we have focussed on flavonoid-rich foods and beverages, we cannot rule out that the observed associations may be due to other constituents found in the whole food or to other uncaptured factors in the meal context. Furthermore, other flavonoid-bearing foods, such a dark chocolate and herbs, and detailed information on factors that influence a foods’ flavonoid content, such as plant variety, growing, storage, processing and cooking methods [50], were not captured in all FFQs. Although we adjusted for a wide range of possible confounders, there is still the possibility of residual confounding from additional unmeasured factors. However, given our detailed and updated adjustment for potential confounders, it is unlikely that these would account fully for the observed results. Furthermore, due to the observational nature of this study, we cannot conclude that observed associations are causal. Findings require replication in other populations; while the NHS and HPFS have high internal validity owing to the health knowledge and commitment to research of the participants [51], these two cohorts are not representative of the general population as they include only nurses or health professionals and participants were mostly Caucasian. Finally, because of the potential for reverse causation to influence these findings, further studies of these relationships are needed.

## Conclusions

Altogether, findings from the present study suggest that not all flavonoid-rich foods are equal. Our novel data suggests that increasing intakes of specific flavonoid-rich foods, namely tea, blueberries, red wine and peppers, even in middle age, may reduce mortality risk.

## Supplementary Information

**Additional file 1:** **Fig. S1.** Flow chart of participants. **Fig. S2.** Time trends in mean intakes of flavonoid-rich foods in the Nurses’ Health Studyand Health Professionals Follow-Up Study. **Table S1.** Associations between all-cause mortality1 and 8-year change in intake of flavonoid-rich foods. 

## Data Availability

Data described in the article, code book, and analytic code will be made available upon request pending approval by the Channing Division of Network Medicine at Brigham and Women’s Hospital and Harvard Medical School. Further information including the procedures to obtain and access data from the Nurses’ Health Study and the Health Professionals Follow-Up Study is described at https://www.nurseshealthstudy.org/researchers (contact e-mail: nhsaccess@channing.harvard.edu) and https://sites.sph.harvard.edu/hpfs/for-collaborators/.

## Abbreviations

BMI: Body mass index
CI: Confidence interval
CVD: Cardiovascular disease
FFQ: Food frequency questionnaire
HPFS: Health Professionals Follow-up Study
HR: Hazard ratio
ICD: International Classification of Diseases
NHS: Nurses’ Health Study

## References

[1] Bondonno NP, Dalgaard F, Kyrø C, Murray K, Bondonno CP, Lewis JR (2019). Flavonoid intake is associated with lower mortality in the Danish Diet Cancer and Health Cohort. Nat Commun.

[2] Bertoia ML, Mukamal KJ, Cahill LE, Hou T, Ludwig DS, Mozaffarian D (2015). Changes in intake of fruits and vegetables and weight change in United States men and women followed for up to 24 years: analysis from three prospective cohort studies. PLoS Med.

[3] Raman G, Avendano EE, Chen S, Wang J, Matson J, Gayer B (2019). Dietary intakes of flavan-3-ols and cardiometabolic health: systematic review and meta-analysis of randomized trials and prospective cohort studies. Am J Clin Nutr.

[4] Ivey KL, Jensen MK, Hodgson JM, Eliassen AH, Cassidy A, Rimm EB (2017). Association of flavonoid-rich foods and flavonoids with risk of all-cause mortality. Br J Nutr.

[5] Parmenter BH, Croft KD, Hodgson JM, Dalgaard F, Bondonno CP, Lewis JR (2020). An overview and update on the epidemiology of flavonoid intake and cardiovascular disease risk. Food Funct.

[6] Williamson G, Kay CD, Crozier A (2018). The bioavailability, transport, and bioactivity of dietary flavonoids: a review from a historical perspective. Compr Rev Food Sci Food Saf.

[7] Williamson G, Kay CD, Crozier A (2018). The bioavailability, transport, and bioactivity of dietary flavonoids: a review from a historical perspective. Compr Rev Food Sci Food Saf.

[8] Willet W, Buzzard I (1998). Foods and Nutrients. Monogr Epidemiol Biostat.

[9] Wilson MM, Reedy J, Krebs-Smith SM (2016). Dietetics. American diet quality: where it is, where it is heading, and what it could be. J Acad Nutr.

[10] Wang DD, Li Y, Bhupathiraju SN, Rosner BA, Sun Q, Giovannucci EL (2021). Fruit and vegetable intake and mortality: results from 2 prospective cohort studies of US men and women and a meta-analysis of 26 cohort studies. Circulation.

[11] Kim K, Vance TM, Chun O (2016). Estimated intake and major food sources of flavonoids among US adults: changes between 1999–2002 and 2007–2010 in NHANES. Eur J Nutr.

[12] McCullough ML, Peterson JJ, Patel R, Jacques PF, Shah R, Dwyer JT (2012). Flavonoid intake and cardiovascular disease mortality in a prospective cohort of US adults. Am J Clin Nutr.

[13] Vogiatzoglou A, Mulligan AA, Lentjes MA, Luben RN, Spencer JP, Schroeter H (2015). Flavonoid intake in European adults (18 to 64 years). PLoS ONE.

[14] Zamora-Ros R, Knaze V, Rothwell JA, Hémon B, Moskal A, Overvad K (2016). Dietary polyphenol intake in Europe: the European Prospective Investigation into Cancer and Nutrition (EPIC) study. Eur J Nutr.

[15] Bondonno NP, Lewis JR, Blekkenhorst LC, Bondonno CP, Shin JH, Croft KD (2020). Association of flavonoids and flavonoid-rich foods with all-cause mortality: The Blue Mountains Eye Study. Clin Nutr.

[16] Bertoia ML, Rimm EB, Mukamal KJ, Hu FB, Willett WC, Cassidy A. Dietary flavonoid intake and weight maintenance: three prospective cohorts of 124 086 US men and women followed for up to 24 years. BMJ. 2016;352:i17. 10.1136/bmj.i17 PMC4730111 26823518

[17] Colditz GA, Manson JE, Hankinson SE (1997). The Nurses' Health Study: 20-year contribution to the understanding of health among women. J Women's Health.

[18] Rimm EB, Giovannucci EL, Willett WC, Colditz GA, Ascherio A, Rosner B (1991). Prospective study of alcohol consumption and risk of coronary disease in men. Lancet.

[19] Cassidy A, Rimm EB, O'Reilly ÉJ, Logroscino G, Kay C, Chiuve SE (2012). Dietary flavonoids and risk of stroke in women. Stroke.

[20] Al-Shaar L, Yuan C, Rosner B, Dean SB, Ivey KL, Clowry CM (2021). Reproducibility and validity of a semiquantitative food frequency questionnaire in men assessed by multiple methods. Am J Epidemiol.

[21] Yuan C, Spiegelman D, Rimm EB, Rosner BA, Stampfer MJ, Barnett JB (2017). Validity of a dietary questionnaire assessed by comparison with multiple weighed dietary records or 24-hour recalls. Am J Epidemiol.

[22] Salvini S, Hunter DJ, Sampson L, Stampfer MJ, Colditz GA, Rosner B (1989). Food-based validation of a dietary questionnaire: the effects of week-to-week variation in food consumption. Int J Epidemiol.

[23] Feskanich D, Rimm EB, Giovannucci EL, Colditz GA, Stampfer MJ, Litin LB (1993). Reproducibility and validity of food intake measurements from a semiquantitative food frequency questionnaire. J Am Diet Assoc.

[24] Rich-Edwards JW, Corsano KA, Stampfer MJ (1994). Test of the national death index and equifax nationwide death search. Am J Epidemiol.

[25] Sun Q, Townsend MK, Okereke OI, Franco OH, Hu FB, Grodstein F (2010). Physical activity at midlife in relation to successful survival in women at age 70 years or older. Arch Intern Med.

[26] Zheng Y, Li Y, Satija A, Pan A, Sotos-Prieto M, Rimm E, et al. Association of changes in red meat consumption with total and cause specific mortality among US women and men: two prospective cohort studies. BMJ. 2019;365:l2110. 10.1136/bmj.l2110 PMC6559336 31189526

[27] Bondonno NP, Bondonno CP, Ward NC, Hodgson JM, Croft KD (2017). The cardiovascular health benefits of apples: Whole fruit vs. isolated compounds. Trends Food Sci Technol.

[28] Wojcicki JM, Heyman MB (2012). Reducing childhood obesity by eliminating 100% fruit juice. Am J Public Health.

[29] Genser D (2008). Food and drug interaction: consequences for the nutrition/health status. Ann Nutr Metab.

[30] Chung M, Zhao N, Wang D, Shams-White M, Karlsen M, Cassidy A (2020). Dose–response relation between tea consumption and risk of cardiovascular disease and all-cause mortality: a systematic review and meta-analysis of population-based studies. Adv Nutr.

[31] Eleftheriou D, Benetou V, Trichopoulou A, La Vecchia C, Bamia C (2018). Mediterranean diet and its components in relation to all-cause mortality: meta-analysis. Brit J Nutr.

[32] Haseeb S, Alexander B, Baranchuk A (2017). Wine and cardiovascular health: a comprehensive review. Circulation.

[33] Rehm J, Mathers C, Popova S, Thavorncharoensap M, Teerawattananon Y, Patra J (2009). Global burden of disease and injury and economic cost attributable to alcohol use and alcohol-use disorders. Lancet.

[34] González R, Ballester I, López-Posadas R, Suárez M, Zarzuelo A, Martinez-Augustin O (2011). Effects of flavonoids and other polyphenols on inflammation. Crit Rev Food Sci Nutr.

[35] Gomes A, Fernandes E, Lima JL, Mira L, Corvo ML (2008). Molecular mechanisms of anti-inflammatory activity mediated by flavonoids. Curr Med Chem.

[36] Landberg R, Sun Q, Rimm EB, Cassidy A, Scalbert A, Mantzoros CS (2011). Selected dietary flavonoids are associated with markers of inflammation and endothelial dysfunction in US women. J Nutr.

[37] Cassidy A, Rogers G, Peterson JJ, Dwyer JT, Lin H, Jacques PF (2015). Higher dietary anthocyanin and flavonol intakes are associated with anti-inflammatory effects in a population of US adults. Am J Clin Nutr.

[38] Mehta AJ, Cassidy A, Litonjua AA, Sparrow D, Vokonas P, Schwartz J (2016). Dietary anthocyanin intake and age-related decline in lung function: longitudinal findings from the VA Normative Aging Study–3. Am J Clin Nutr.

[39] Garcia-Larsen V, Thawer N, Charles D, Cassidy A, Van Zele T, Thilsing T (2018). Dietary intake of flavonoids and ventilatory function in European adults: A GA2LEN study. Nutrients.

[40] Bondonno NP, Parmenter BH, Dalgaard F, Murray K, Rasmussen DB, Kyrø C, et al. Flavonoid intakes inversely associate with COPD in smokers. Eur Respir J. 2022;60(2):2102604. 10.1183/13993003.02604-2021 PMC9363846 35058251

[41] Ayaz M, Sadiq A, Junaid M, Ullah F, Ovais M, Ullah I (2019). Flavonoids as prospective neuroprotectants and their therapeutic propensity in aging associated neurological disorders. Front Aging Neurosci.

[42] de Andrade Teles RB, Diniz TC, Costa Pinto TC, de Oliveira Júnior RG, Gama e Silva M, de Lavor ÉM, et al. Flavonoids as therapeutic agents in Alzheimer’s and Parkinson’s diseases: a systematic review of preclinical evidences. Oxid Med Cell Longev. 2018;2018:7043213. 10.1155/2018/7043213 PMC5971291 29861833

[43] Bondonno CP, Bondonno NP, Dalgaard F, Murray K, Gardener SL, Martins RN (2021). Flavonoid intake and incident dementia in the Danish Diet, Cancer, and Health cohort. Alzheimer's Dement: Transl Res Clin Interv.

[44] Gao X, Cassidy A, Schwarzschild M, Rimm EB, Ascherio A (2012). Habitual intake of dietary flavonoids and risk of Parkinson disease. Neurology.

[45] Shishtar E, Rogers GT, Blumberg JB, Au R, Jacques PF (2020). Long-term dietary flavonoid intake and risk of Alzheimer disease and related dementias in the Framingham Offspring Cohort. Am J Clin Nutr.

[46] Holland TM, Agarwal P, Wang Y, Leurgans SE, Bennett DA, Booth SL (2020). Dietary flavonols and risk of Alzheimer dementia. Neurology.

[47] Gao S, Nguyen JT, Hendrie HC, Unverzagt FW, Hake A, Smith-Gamble V (2011). Accelerated weight loss and incident dementia in an elderly African-American cohort. J Am Geriatr Soc.

[48] Fávaro-Moreira NC, Krausch-Hofmann S, Matthys C, Vereecken C, Vanhauwaert E, Declercq A (2016). Risk factors for malnutrition in older adults: a systematic review of the literature based on longitudinal data. Adv Nutr.

[49] Kai K, Hashimoto M, Amano K, Tanaka H, Fukuhara R, Ikeda M (2015). Relationship between eating disturbance and dementia severity in patients with Alzheimer’s disease. PLoS ONE.

[50] Perez-Jimenez J, Neveu V, Vos F, Scalbert A (2010). Systematic analysis of the content of 502 polyphenols in 452 foods and beverages: an application of the phenol-explorer database. J Agric Food Chem.

[51] Bao Y, Bertoia ML, Lenart EB, Stampfer MJ, Willett WC, Speizer FE (2016). Origin, methods, and evolution of the three Nurses’ Health Studies. Am J Public Health.

