# Supplementary material for: Change in habitual intakes of flavonoid-rich foods and mortality in US males and females
Source: BMC Med. 2023 May 12;21:181. doi: 10.1186/s12916-023-02873-z (PMC10182674; doi:10.1186/s12916-023-02873-z)
Supplement: Supplementary file 1 — Additional file 1: Fig. S1. Flow chart of participants. Fig. S2. Time trends in mean intakes of flavonoid-rich foods in the Nurses’ Health Studyand Health Professionals Follow-Up Study. Table S1. Associations between all-cause mortality1 and 8-year change in intake of flavonoid-rich foods. [file 12916_2023_2873_MOESM1_ESM.docx]

**Change in habitual intakes of flavonoid-rich foods and mortality in US men and women**

Nicola P. Bondonno, Lydia Liu, Yan Zheng, Kerry Ivey, Walter C. Willett, Meir J. Stampfer, Eric B. Rimm, Aedín Cassidy

**Online Data Supplement**


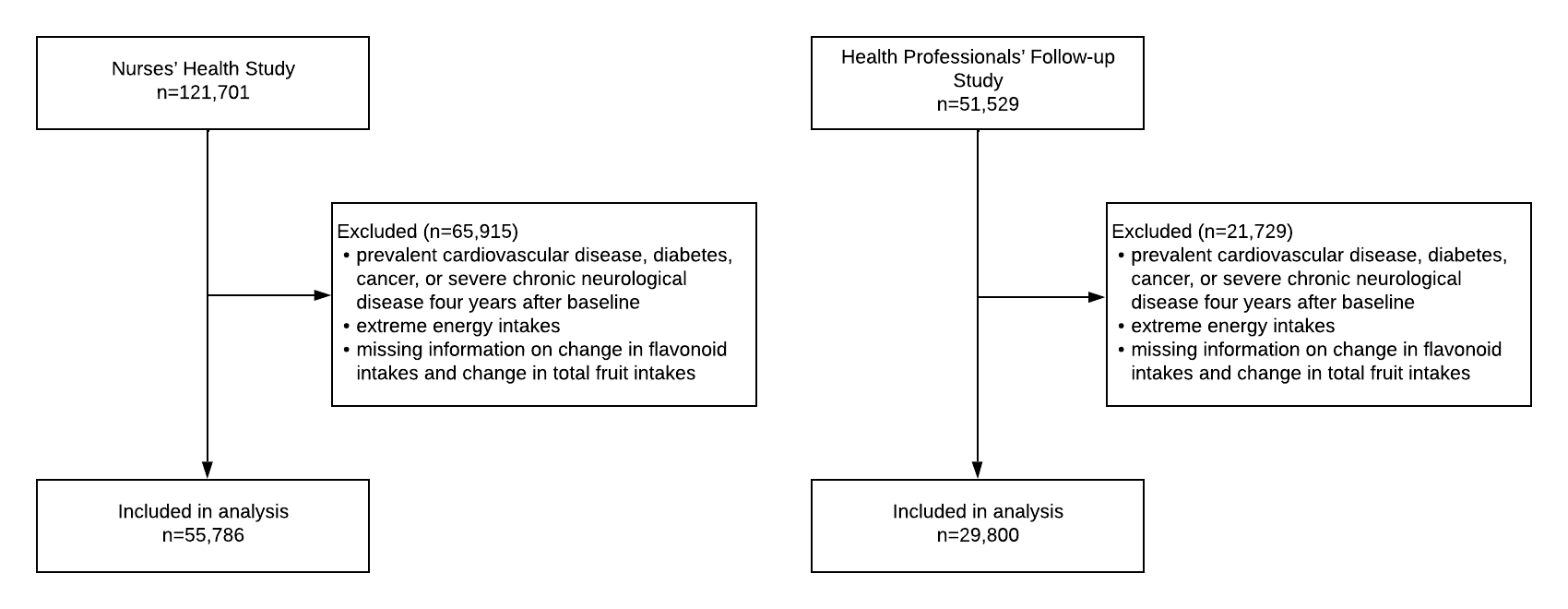


**Fig. S1.** Flow chart of participants.


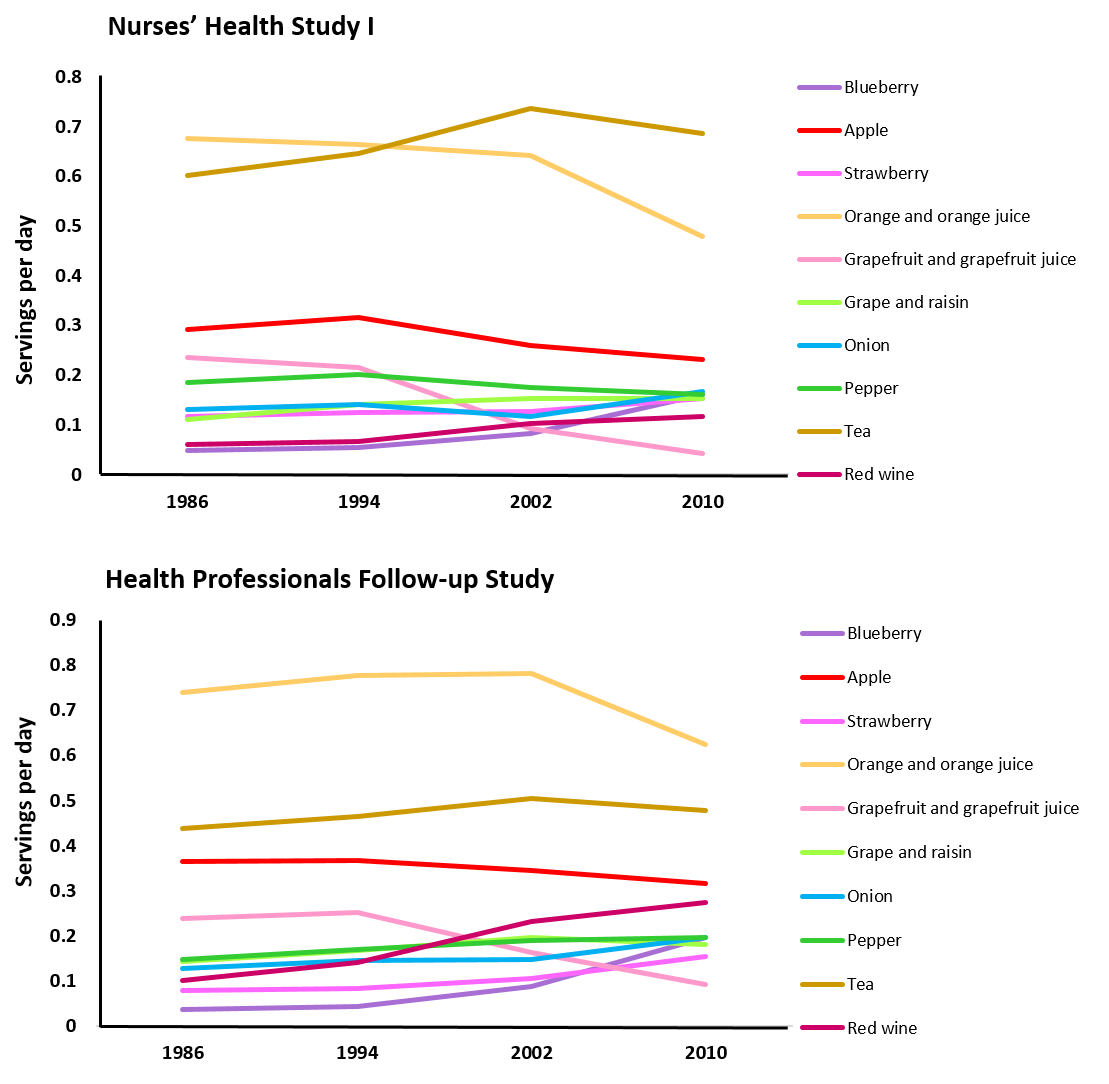


**Fig. S2.** Time trends in mean intakes of flavonoid-rich foods in the Nurses’ Health Study (1986-2010) and Health Professionals Follow-Up Study (1986-2010).

| **Table S1.** Associations between all-cause mortality (Hazard ratios, 95% confidence intervals)^1^ and 8-year change in intake of flavonoid-rich foods | | | | | | | | | |
| --- | --- | --- | --- | --- | --- | --- | --- | --- | --- |
|  | 8-year change in intake levels, serving/wk | | | | | | |  |  |
|  |  | Decrease |  |  |  | Increase |  |  |  |
|  | ≥2 | 1-1.99 | 0.5-0.99 | No change | ≥2 | 1-1.99 | 0.5-0.99 |  | Every  3.5 servings/wk change^3^ |
| ***Blueberry*** |  |  |  |  |  |  |  |  |  |
| NHS |  |  |  |  |  |  |  |  |  |
| Model 1^2^ |  | 1.40 (1.24, 1.60) | 1.10 (0.99, 1.22) | 1.00 | 0.88 (0.80, 0.96) | 0.86 (0.81, 0.91) |  |  | 0.78 (0.73, 0.82) |
| HPFS |  |  |  |  |  |  |  |  |  |
| Model 1^2^ |  | 1.13 (0.94, 1.36) | 1.16 (0.96, 1.41) | 1.00 | 0.94 (0.83, 1.06) | 0.94 (0.87, 1.01) |  |  | 0.92 (0.87, 0.98) |
| ***Apple*** |  |  |  |  |  |  |  |  |  |
| NHS |  |  |  |  |  |  |  |  |  |
| Model 1^2^ | 1.05 (1.00, 1.11) | 1.06 (0.87, 1.30) | 1.11 (1.00, 1.22) | 1.00 | 1.02 (0.90, 1.16) | 1.04 (0.84, 1.28) | 0.98 (0.93, 1.02) |  | 0.94 (0.91, 0.97) |
| HPFS |  |  |  |  |  |  |  |  |  |
| Model 1^2^ | 1.04 (0.97, 1.10) | 1.08 (0.90, 1.31) | 1.16 (0.98, 1.38) | 1.00 | 1.11 (0.90, 1.36) | 0.87 (0.71, 1.07) | 0.98 (0.92, 1.03) |  | 0.94 (0.91, 0.97) |
| ***Orange and orange juice*** | |  |  |  |  |  |  |  |  |
| NHS |  |  |  |  |  |  |  |  |  |
| Model 1^2^ | 1.02 (0.98, 1.07) | 0.96 (0.89, 1.05) | 0.89 (0.82, 0.96) | 1.00 | 1.09 (0.99, 1.19) | 0.98 (0.90, 1.08) | 1.07 (1.02, 1.11) |  | 1.01 (0.99, 1.02) |
| HPFS |  |  |  |  |  |  |  |  |  |
| Model 1^2^ | 1.03 (0.97, 1.09) | 0.90 (0.80, 1.01) | 1.00 (0.89, 1.13) | 1.00 | 0.91 (0.80, 1.04) | 0.88 (0.78, 0.99) | 1.03 (0.97, 1.09) |  | 0.99 (0.97, 1.00) |
| ***Tea*** |  |  |  |  |  |  |  |  | ^3^1 serving/day |
| NHS |  |  |  |  |  |  |  |  |  |
| Model 1^2^ | 1.00 (0.95, 1.05) | 0.89 (0.78, 1.00) | 1.02 (0.93, 1.12) | 1.00 | 0.90 (0.82, 1.00) | 0.78 (0.68, 0.91) | 0.89 (0.86, 0.93) |  | 0.95 (0.93, 0.97) |
| HPFS |  |  |  |  |  |  |  |  |  |
| Model 1^2^ | 1.02 (0.96, 1.09) | 0.84 (0.70, 1.00) | 1.01 (0.89, 1.15) | 1.00 | 0.96 (0.84, 1.08) | 0.85 (0.71, 1.02) | 0.94 (0.89, 1.00) |  | 0.96 (0.93, 0.99) |
| ***Grapefruit and grapefruit juice*** | | | |  |  |  |  |  |  |
| NHS |  |  |  |  |  |  |  |  |  |
| Model 1^2^ | 0.92 (0.86, 0.99) | 0.89 (0.78, 1.01) | 0.97 (0.91, 1.03) | 1.00 | 1.16 (1.03, 1.31) | 1.13 (0.94, 1.37) | 1.01 (0.94, 1.09) |  | 1.05 (1.01, 1.09) |
| HPFS |  |  |  |  |  |  |  |  |  |
| Model 1^2^ | 0.99 (0.91, 1.06) | 0.97 (0.81, 1.15) | 1.00 (0.91, 1.10) | 1.00 | 1.20 (1.04, 1.38) | 0.91 (0.73, 1.11) | 1.04 (0.97, 1.12) |  | 1.03 (0.99, 1.07) |
| ***Red wine*** |  |  |  |  |  |  |  |  |  |
| NHS |  |  |  |  |  |  |  |  |  |
| Model 1^2^ |  | 1.00 (0.91, 1.11) | 0.92 (0.80, 1.05) | 1.00 | 0.74 (0.63, 0.86) | 0.75 (0.69, 0.81) |  |  | 0.87 (0.83, 0.91) |
| HPFS |  |  |  |  |  |  |  |  |  |
| Model 1^2^ |  | 1.07 (0.97, 1.17) | 1.21 (1.04, 1.41) | 1.00 | 0.84 (0.71, 0.97) | 0.82 (0.76, 0.87) |  |  | 0.91 (0.88, 0.94) |
| ***Strawberry*** |  |  |  |  |  |  |  |  |  |
| NHS |  |  |  |  |  |  |  |  |  |
| Model 1^2^ |  | 1.14 (1.04, 1.24) | 1.20 (1.11, 1.30) | 1.00 | 1.03 (0.94, 1.15) | 1.01 (0.96, 1.07) |  |  | 0.93 (0.88, 0.98) |
| HPFS |  |  |  |  |  |  |  |  |  |
| Model 1^2^ |  | 0.97 (0.85, 1.12) | 1.20 (1.04, 1.39) | 1.00 | 1.06 (0.93, 1.21) | 1.08 (1.00, 1.16) |  |  | 1.04 (0.98, 1.11) |
| ***Onion*** |  |  |  |  |  |  |  |  |  |
| NHS |  |  |  |  |  |  |  |  |  |
| Model 1^2^ |  | 0.91 (0.87, 0.96) | 0.93 (0.87, 0.99) | 1.00 | 1.10 (0.97, 1.25) | 1.03 (0.95, 1.10) |  |  | 1.09 (1.05, 1.14) |
| HPFS |  |  |  |  |  |  |  |  |  |
| Model 1^2^ |  | 0.99 (0.90, 1.08) | 0.97 (0.85, 1.11) | 1.00 | 0.96 (0.86, 1.07) | 1.03 (0.97, 1.10) |  |  | 1.03 (0.98, 1.08) |
| ***Peppers*** | |  |  |  |  |  |  |  |  |
| NHS |  |  |  |  |  |  |  |  |  |
| Model 1^2^ |  | 1.17 (1.10, 1.25) | 1.24 (1.14, 1.35) | 1.00 | 1.01 (0.89, 1.14) | 0.88 (0.83, 0.93) |  |  | 0.82 (0.78, 0.86) |
| HPFS |  |  |  |  |  |  |  |  |  |
| Model 1^2^ |  | 1.04 (0.95, 1.13) | 1.02 (0.88, 1.18) | 1.00 | 1.02 (0.88, 1.19) | 0.92 (0.87, 0.99) |  |  | 0.93 (0.88, 0.97) |
| ***Grape and raisin*** | |  |  |  |  |  |  |  |  |
| NHS |  |  |  |  |  |  |  |  |  |
| Model 1^2^ |  | 1.00 (0.95, 1.05) | 1.01 (0.93, 1.10) | 1.00 | 0.93 (0.86, 1.01) | 0.90 (0.85, 0.95) |  |  | 0.94 (0.91, 0.97) |
| HPFS |  |  |  |  |  |  |  |  |  |
| Model 1^2^ |  | 1.01 (0.93, 1.09) | 0.99 (0.87, 1.13) | 1.00 | 1.10 (0.96, 1.25) | 0.96 (0.90, 1.02) |  |  | 0.96 (0.93, 1.00) |
| ^1^ All hazard ratios (95% confidence intervals) were calculated using Cox proportional hazard models. HPFS, Health Professionals Follow-up Study; NHS, Nurses’ Health Study.  ^2^ Model 1 was adjusted for age and baseline intakes of the exposure variable.  ^3^ Except for tea, where the hazard ratio (95%CI) is presented for an 8-year change in intake of 1 serve per day. | | | | | | | | | |
